# Cardiac cephalalgia: a narrative review and ICHD-3 criteria evaluation

**DOI:** 10.1186/s10194-022-01508-7

**Published:** 2022-10-20

**Authors:** María Pilar Navarro-Pérez, Elena Bellosta-Diago, Jes Olesen, Sonia Santos-Lasaosa

**Affiliations:** 1 Neurology Department, Hospital Clínico Universitario Lozano Blesa, San Juan Bosco 15, 50009 Saragossa, Spain; 2 Aragon Institute for Health Research (IIS Aragón), Saragossa, Spain; 3 Danish Headache Center, Department of Neurology, Rigshospitalet-Glostrup, University of Copenhagen, Copenhagen, Denmark

**Keywords:** Secondary headache, Myocardial ischemia, Myocardial infarction, Headache, Exertional headache

## Abstract

**Background:**

Cardiac cephalalgia is an unusual condition that occurs during an episode of myocardial ischemia. Information about cardiac cephalalgia is scarce and its characteristics and physiopathology remain unclear. Our aim is to provide a narrative review of clinical characteristics and physiopathology of cardiac cephalalgia and to evaluate the current diagnostic criteria.

**Methods:**

A search through PubMed was undertaken for studies on cardiac cephalalgia published until 20^th^ September 2022. We summarized the literature and provide a comprehensive review of the headache characteristics and possible mechanisms. We also evaluated current International Classification of Headache Disorders third edition diagnostic criteria based on prior reported cases.

**Results:**

In total, 88 cases were found. Headache characteristics were variable. Occipital location and throbbing pain were the most frequently reported. Headache was accompanied in most cases by cardiac symptoms. Criterion B was fulfilled by 98% of cases, criterion C1 by 72%, and criteria C2a and C2b by 37 and 93.2%, respectively. Regarding headache features described in diagnostic criterion C3, ‘moderate to severe intensity’, ‘accompanied by nausea’, ‘not accompanied by photophobia or phonophobia’ and ‘aggravated by exertion’, were reported in 75, 31, 55 and 55% of cases, respectively.

**Conclusion:**

Cardiac cephalalgia characteristics are variable and the headache features described in the diagnostic criterion C3 might not be adequate. Given that cardiac cephalalgia can be the manifestation of a life-threatening condition it is important to increase the knowledge about this entity.

**Supplementary Information:**

The online version contains supplementary material available at 10.1186/s10194-022-01508-7.

## Introduction

Comorbidities of primary headache disorders include cardiovascular conditions and ischemic heart disease prevalence is higher in patients with primary headache that in general population (7% vs. 2.6%) [1]. Nonetheless, headache itself can be an infrequent presentation of myocardial ischemia. With regard to previous studies that evaluated presenting symptoms of myocardial ischemia, it has been reported that only 5.2 – 6% of patients with acute myocardial ischemia presented headache as an accompanying symptom [2, 3]. In 3.5% of cases headache was the main pain location [2] but headache was not reported as the sole symptom by any patient [3].

Cardiac cephalalgia is an unusual condition that occurs in relation to an episode of myocardial ischemia. This term was first proposed by Lipton et al. in 1997 [4] and, since then, several case reports have been published. Nevertheless, prevalence of cardiac cephalalgia is unknown, information about cardiac cephalalgia characteristics is scarce and its physiopathology is yet to be elucidated.

Since cardiac cephalalgia can be the main or even the only clinical manifestation of a myocardial infarction, a life-threatening condition, recognizing this entity is of great importance. Therefore, our aim is to review the clinical characteristics of cardiac cephalalgia and the proposed pathogenic mechanisms and to evaluate the current International Classification of Headache Disorders third edition (ICHD-3) [5] diagnostic criteria on the basis of previous case reports.

## Methods

We performed a literature search in Pubmed database on 20 September 2022. We used the following search lines: “Cardiac”[Title/Abstract] and “Cephalgia”[Title/Abstract], “Cardiac”[Title/Abstract] and “Cephalalgia”[Title/Abstract], “Acute myocardial infarction”[Title/Abstract] and “Headache”[Title/Abstract], “Myocardial Infarction”[MeSH] and “Headache”[MeSH], “Myocardial Ischemia”[MeSH]) and “Headache”[MeSH], “Acute Coronary Syndrome”[MeSH] and “Headache”[MeSH], “Angina”[MeSH] and “Headache”[MeSH]. Only manuscripts written in English and Spanish were included. We applied no time limits. We selected all publications relevant to our topic and we also reviewed the references of these papers in order to find other useful material to our review.

**Fig. 1:**
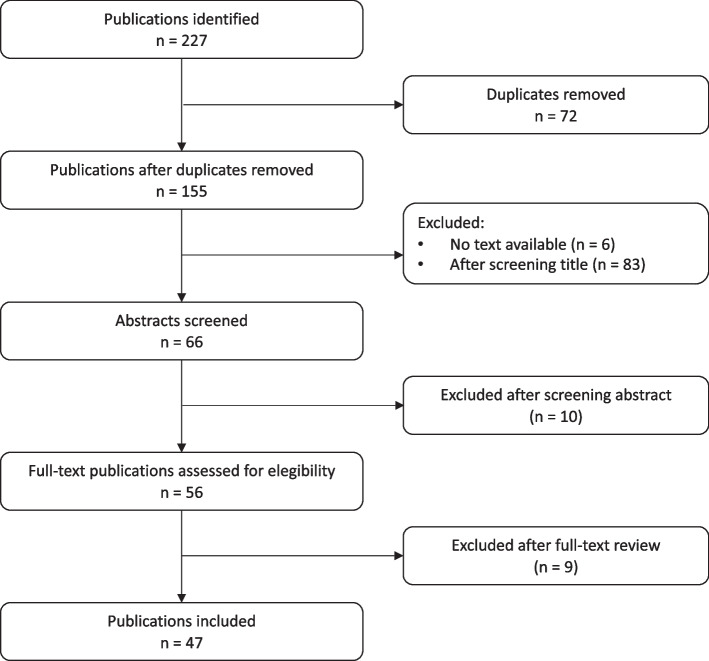
Flow chart of literature search

We identified 227 records of which, after deduplication, 155 remained for further analysis. A total of 66 articles were screened by abstract. After a further screening process, 50 publications were finally included, three of them from the review of the references of selected papers (Fig. 1).

For the evaluation of the current diagnostic criteria, we calculated the proportion of cases that fulfilled the ICHD-3 diagnostic criteria B, C1, C2, C3 and C4 in relation to the total of cases. Non-specified cases were classified as ‘no’.

## Results

In 1997 Lipton et al. were the first to coin the term cardiac cephalalgia [4]. They reported two patients with exertional headache in relation to myocardial ischemia and reviewed five previously published cases. Although initially in this first description cardiac cephalalgia was exclusively an exertional headache, in subsequent publications patients with onset at rest were also reported.

Cardiac cephalalgia was introduced in the second edition of the International Classification of Headache Disorders (ICHD-2) as a secondary headache in the category of ‘headache attributed to disorder of homeostasis’ [6]. ICH-2 diagnostic criteria can be seen in Table 1.

**Table 1:** ICHD-2 diagnostic criteria for cardiac cephalalgia [6]

10.6 Cardiac Cephalalgia
A. Headache, which may be severe, aggravated by exertion and accompanied by nausea and fulfilling criteria C and D
B. Acute myocardial ischaemia has occurred
C. Headache develops concomitantly with acute myocardial ischaemia
D. Headache resolves and does not recur after effective medical therapy for myocardial ischemia or coronary revascularisation

In 2004, Chen et al. [7] performed a literature review and evaluated ICHD-2 diagnostic criteria based on 22 prior reported cases. All cases fulfilled ICHD-2 criteria B and C. However, regarding headache characteristics described in criterion A, severe intensity was reported by 59% of cases, aggravation by exertion by 50% and, interestingly, only 23% of cases reported nausea. Moreover, resolution of headache in criterion D was fulfilled by 83% but in some patients headache recurred.

In the ICHD-3 [5], cardiac cephalalgia is described as a migraine-like headache, usually but not always aggravated by exercise, occurring during an episode of myocardial ischemia. Table 2 shows ICHD-3 diagnostic criteria for cardiac cephalalgia.

**Table 2:** ICHD-3 diagnostic criteria for cardiac cephalalgia [5]

10.6 Cardiac Cephalalgia
A. Any headache fulfilling criterion C
B. Acute myocardial ischaemia has been demonstrated
C. Evidence of causation demonstrated by at least two of the following:
1. headache has developed in temporal relation to the onset of acute myocardial ischaemia
2. Either or both of the following:
a) headache has significantly worsened in parallel with worsening of the myocardial ischaemia
b) headache has significantly improved or resolved in parallel with improvement in or resolution of the myocardial ischaemia
3. headache has at least two of the following four characteristics:
a) moderate to severe intensity
b) accompanied by nausea
c) not accompanied by photophobia or phonophobia
d) aggravated by exertion
4. headache is relieved by nitroglycerine or derivatives of it
D. Not better accounted for by another ICHD-3 diagnosis

### Patient characteristics

We found a total of 88 cases of cardiac cephalalgia, 58 from case reports [7–53] (a complete description of these cases can be seen in supplementary material) and 30 patients from a recent observational study [54]. Among case reports, mean age at onset was 62.3 years (± 13.1) and most patients were males (*n* = 36; 62.1%). These data are in line with the study from Xu et al. that reported a mean age of 64.6 years (± 11.9) and a predominance of male patients (53.3%) [54]. History of cardiovascular risk factors was common. It was present in 41 (70.7%) of the case reports and in all the patients from the observational study [54]. History of headache was reported only in 5 patients (8.6%). Nevertheless, it was not specified in most case reports and was not assessed in the observational study [54] (Table 3).

**Table 3:** Demographics and headache characteristics of patients with cardiac cephalalgia from our review (*n* = 88)

	n (%)
Sex	Male 52 (59.1%)
	Female 36 (40.9%)
Cardiovascular risk factors	Yes 71 (80.7%)
	No 9 (10.2%)
	NS 8 (9.1%)
Headache history	Yes 5 (5.7%)
	No 17 (19.3%)
	NS 66 (75%)
Headache location	
Occipital	19 (21.6%)
Parietal	3 (3.4%)
Temporal	13 (14.8%)
Frontal	12 (13.6%)
Vertex	2 (2.3%)
Holocranial	11 (12.5%)
Eye	2 (2.3%)
Bregma	1 (1.1%)
Right lateralized headache	1 (1.1%)
Two or more regions	14 (15.9%)
NS	10 (11.3%)

*NS* not specified

### Headache characteristics

**Fig. 2:**
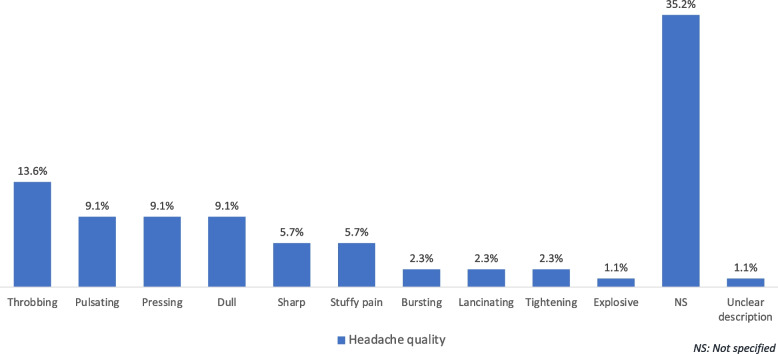
Headache quality reported by patients with cardiac cephalalgia

Regarding headache features, location of pain was variable and occipital pain was the most frequently reported among previous case reports (*n* = 16, 27,6%) [7–53]. Nevertheless, pain located in the frontotemporal region was the most frequent in the study by Xu et al. (*n* = 14, 46.6%) [54]. Bilateral headache was reported in 24 patients (41.4%) and unilateral headache in 7 (12.1%) of the case reports (Table 3). Headache quality was also very variable. Among case reports, throbbing (*n* = 8, 13.8%) and pressing pain (*n* = 7, 12.1%) were the most frequent but in one-half of the cases headache quality was not specified. Xu et al. found that pulsating quality was the most frequent (33.3%) followed by dull (16.7%) and stuffy pain (16.7%) [54] (Fig. 2).

In total, more than half of the 88 cases reported severe intensity (*n* = 51, 57.9%), moderate intensity was described by 15 cases (17%), mild intensity by 6 (6.8%) and variable intensity by 3 (3.4%). In 13 patients (14.7%) headache intensity was not specified. Headache was accompanied by nausea in 28 cases (31.8%), was not accompanied by photophobia or phonophobia in 49 patients (55.7%) and was aggravated or induced by exertion in 49 (55.7%). It is also interesting that two cases reported tearing and one case reported rhinorrhoea [54].

### Myocardial ischemia features

In 21 patients from the case reports headache was the only symptom of the myocardial ischemia [7–53]. Nevertheless, in line with the results from the study from Xu et al. [54], most patients presented other cardiac symptoms including typical angina manifestations (Table 4). 23 patients underwent stress test, which was positive in most cases and in 17 patients headache was reproduced during the stress test (Table 4). Coronary angiography was performed in 78 patients (88.6%) and most patients presented severe coronary disease. Single-vessel disease was reported in 21 cases (23.9%), double-vessel disease in 15 cases (17%), triple-vessel or multivessel disease in 39 cases (44.3%), vasospasm was found in one case [35] and two cases presented normal coronary arteries [30, 35]. Although the affected vascular territory was variable, the left anterior descending artery (*n* = 27, 30.7%) and the right coronary artery (*n* = 26, 29.5%) were the most frequently affected (Table 4). 22 patients (42.3%) were treated by percutaneous transluminal coronary angioplasty, 12 cases (23.1%) required coronary artery bypass and 12 patients (23.1%) received medical treatment. Headache improved or resolved in all cases after treatment of the myocardial ischemia. Although the follow-up period was variable, 37 patients (42%) experienced complete resolution, 33 (37.5%) improved, five patients (5.7%) experienced headache recurrence, eight patients (9.1%) died in the acute phase or during the follow-up period and in five cases (5.7%) outcome was not specified. Four of the patients with headache recurrence experienced simultaneous myocardial ischemia recurrence [11, 15, 18, 42].

**Table 4:** Summary of cardiac symptoms, stress test results and coronary arteries affected in patients with cardiac cephalalgia from our review (*n* = 88)

	n (%)
Cardiac symptoms	
Chest pain or chest tightness	48 (54.5%)
Left arm pain	6 (6.8%)
Sweating	23 (26.1%)
Dyspnea	10 (11.3%)
Palpitations	5 (5.7%)
Cardiac arrest	4 (4.5%)
Stress test	
Positive	21 (23.9%)
Negative	2 (2.3%)
NS	65 (73.8%)
Affected coronary arteries	
RCA	26 (29.5%)
CXA	17 (19.3%)
LAD	27 (30.7%)
PDA	1 (1.1%)
Left main trunk	2 (2.3%)
NS	47 (53.4%)

*NS* Not specified, *RCA* Right coronary artery, CXA Circumflex artery, *LAD* Left anterior descending artery, *PDA* Posterior descending artery

### ICHD-3 diagnostic criteria evaluation

Criterion B ‘Acute myocardial ischemia has been demonstrated’ was fulfilled by 87 cases (98.9%). In one case of headache with and without exertion relieved by nitrates myocardial ischemia was not demonstrated because patient died suddenly without electrocardiogram and cardiac enzymes determination [25] (Table 5).

**Table 5:** Application of ICHD-3 diagnostic criteria for cardiac cephalalgia in patients with cardiac cephalalgia from our review

Criteria	n	%
B. Acute myocardial ischemia has been demonstrated	87/88	98.9%
C1. Headache has developed in temporal relation to the onset of acute myocardial ischaemia	64/88	72.7%
C2a. Headache has significantly worsened in parallel with worsening of the myocardial ischaemia	33/88	37.5%
C2b. Headache has significantly improved or resolved in parallel with improvement in or resolution of the myocardial ischaemia	82/88	93.2%
C3a. moderate to severe intensity	66/88	75%
C3b. accompanied by nausea	28/88	31.8%
C3c. not accompanied by photophobia or phonophobia	49/88	55.7%
C3d. aggravated by exertion	49/88	55.7%
C4. Headache is relieved by nitroglycerine or derivatives of it	40/40	100%

*ICHD-3* international classification of headache disorders third edition

To evaluate criterion C1 `Headache has developed in temporal relation to the onset of acute myocardial ischaemia’ it is necessary to observe the temporal relationship of headache and cardiac symptoms but it can only be confirmed if we observe the onset of headache during a stress test. We considered that 34 patients from the case reports met this criterion. 17 patients reported headache onset in relation to onset of cardiac symptoms and in 17 cases stress tests confirmed the temporal relationship between myocardial ischemia and headache. All 30 patients from the study from Xu et al. met this criterion [54] (Table 5).

Regarding criterion C2 most patients (93.2%) fulfilled criterion C2b ‘Headache has significantly improved or resolved in parallel with improvement in or resolution of the myocardial ischaemia’. However, evaluate criterion C2a is difficult because it is hard to determine the worsening of myocardial ischemia. For this reason, we considered that only three cases who presented worsening of headache in relation to confirmed worsening of the myocardial ischemia during the coronarography met criterion C2a [13, 14, 52]. Xu et al. reported that all 30 patients met this criterion [54] (Table 5).

Criterion C3 is the criterion that describes headache characteristics. Criterion C3a ‘moderate to severe intensity’ was met by 75%, criterion C3b ‘accompanied by nausea’ by 31.8%, criterion C3c ‘not accompanied by photophobia or phonophobia’ by 55.7% and criterion C3d ‘aggravated by exertion’ by 55.7%. Considering these findings, nausea is not a frequent accompanying symptom in cardiac cephalalgia. This finding is in line with the results of the literature review by Chen et al. [6] that found accompanying nausea only in 23% of cases and with the study from Xu et al. [54] that reported that 36.6% of patients fulfilled this criterion.

Finally, among those cases that reported nitrates administration, Criterion C4 ‘Headache is relieved by nitroglycerine or derivatives of it’ was fulfilled by 100% of cases. However, it must be considered that in 36 case reports the use of nitrates was not specified and that in the study by Xen et al. all 12 patients did not receive nitrates [54].

Taking into account the present evaluation and in order to improve ICHD-3 diagnostic criteria, we might suggest removing criterion C3b ‘accompanied by nausea’ given that it was the least commonly fulfilled criterion.

### Pathophysiology

Even though the pathophysiology of cardiac cephalalgia is not known, four hypotheses have been proposed to illustrate its mechanisms [7, 53–56].

The first hypothesis states that cardiac cephalalgia could be the result of the convergence of afferent autonomic visceral fibers, which carry nociceptive cardiac stimuli, and somatic sensory fibers onto a common pool of spinothalamic tract (STT) and spinoreticular tract (SRT) neurons. Thus, according to this hypothesis, cardiac cephalalgia would be a referred pain produced because the brain interprets that cardiac pain is originated in the somatic structures [57–59].

Foreman et al. [58] proposed that ‘typical angina’ and ‘atypical angina’ would have different mechanisms. In the first scenario, sympathetic cardiac afferents may converge onto STT and SRT neurons with the somatic stimuli from the chest and upper arm in the upper thoracic and cervical spinal segments (T1-T5 and C5-C6). In contrast, angina associated to referred pain to the neck and jaw might be mediated by cardiac vagal afferents that predominantly terminate in the nucleus tractus solitarius and then project to STT neurons in the C1-C2 segment. Cervical and trigeminal afferents converge in the trigeminocervical complex which is constituted by C1 and C2 segments and the trigeminal nucleus caudalis [60, 61]. Therefore, vagal cardiac afferents could activate trigeminal neurons and produce referred pain in the cervical and trigeminal territory.

**Fig. 3:**
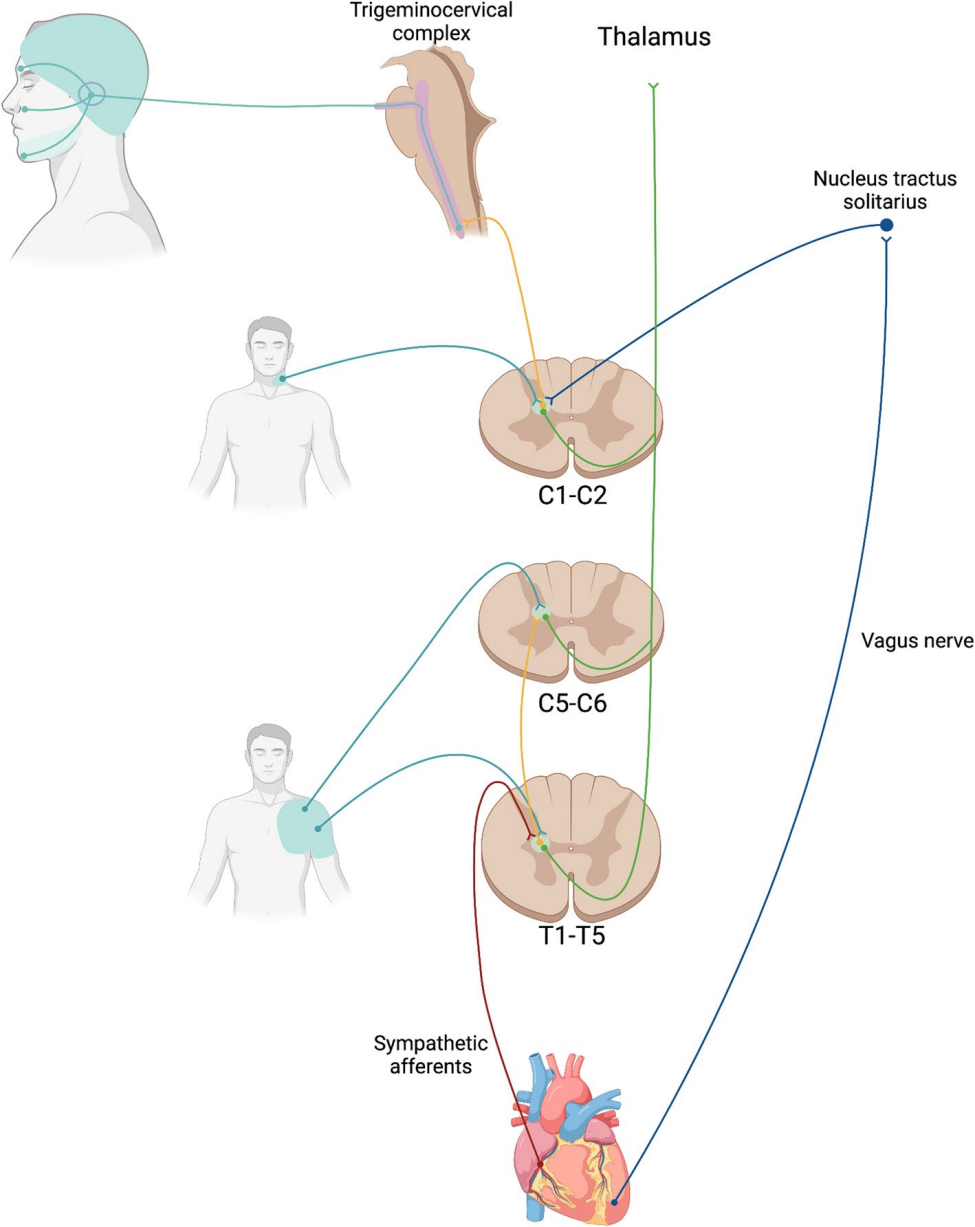
Convergence theory of cardiac cephalalgia. Adapted from Foreman [58]. Adapted with permission from John Wiley and Sons. Figure legend: Sympathetic cardiac afferents converge in the spinothalamic tract (STT) neurons in the T1-T5 and C5-C6 segments with somatic fibers from the chest and upper arm respectively. Vagal afferents primarily terminate in the nucleus tractus solitarius and then project to the STT neurons located in C1-C2 segments. These segments also receive nociceptive information from the neck and could also receive nociceptive input from the trigeminal region through the trigeminocervical complex. However, C1-C2 segments do not receive significant stimuli from sympathetic afferents

The concentration of cardiac afferent autonomic visceral fibers can vary for each cardiac region [62]. Although the anatomy and physiology of this fibers are not yet fully understood, it is thought that a greater concentration of sympathetic cardiac afferent fibers is primarily located in the anterior part of the left ventricle whereas parasympathetic afferent fibers mainly innervate the inferior-posterior wall of the left ventricle [58]. Since the anterior portion of the left ventricle is mainly perfused by the left anterior descending artery, whereas the posterior and inferior walls are principally supplied by the right coronary artery and the circumflex artery, the predominant activation of sympathetic or parasympathetic fibers may be determined by the artery responsible for the myocardial ischemia [58, 63]. Therefore, cardiac cephalalgia might depend on the area of the heart in which myocardial ischemia occurs. Figure 3 shows a representation of the convergence theory of cardiac cephalalgia.

The second theory states that the abrupt decrease of cardiac output during myocardial ischemia would cause a rising of pressure in the left atrium and left ventricle hindering cerebral venous return. Hence, cardiac cephalalgia would be the result of an increased intracranial pressure secondary to a reduction of cerebral venous drainage [7, 53–56].

The third hypothesis suggests that the release of inflammatory mediators during cardiac ischemia including bradykinin, serotonin, substance P and histamine could cause vasodilation in brain arteries leading to headache [7, 53–56].

Finally, Wang et al. [46] proposed a fourth hypothesis. They reported a case of cardiac cephalalgia with cortical cerebral hypoperfusion confirmed by the perfusion-weighted images of brain magnetic resonance (MR) imaging and with normal MR angiography during the headache attack. Therefore, they deduced that cardiac cephalalgia might be the result of the vasoconstriction of small intracranial arteries caused by the activation of the sympathetic system by myocardial ischemia. They also proposed that cortical cerebral hypoperfusion may provoke cortical spreading depression which could additionally contribute to headache.

### Limitations and future perspective

We provide an updated overview on cardiac cephalalgia from the physiopathology to the diagnostic criteria. The major limitation of our review is that the description of headache characteristics of cardiac cephalagia in a high proportion of previous reports were scarce and that there were a large number of terms to describe headache quality. Another limitation is that headache history was rarely reported. Thus, patients with a worsening of a preexisting headache may have been misdiagnosed as cardiac cephalalgia. To further elucidate the characteristics of cardiac cephalalgia and its pathophysiology it would be of great interest to conduct further studies with standardized data collection of patients with demonstrated myocardial ischemia in order to investigate cardiac cephalalgia prevalence and characteristics.

## Conclusion

Cardiac cephalalgia characteristics are variable and the proposed headache features in the diagnostic criterion C3 might not be adequate, especially nausea which was the least commonly met criterion. Given that it can be the manifestation of a life-threatening condition it is important to increase the knowledge about this entity. Further prospective studies are warranted to assess its prevalence and to describe in detail its clinical features in order to better understand and manage patients with cardiac cephalalgia.

## Supplementary Information

**Additional file1.** Cardiac cephalalgia case reports. Summary of data from previous case reports of cardiac cephalalgia.

## Data Availability

Not applicable.

## Abbreviations

ICHD-3: International Classification of Headache Disorders third edition
ICHD-2: International Classification of Headache Disorders second edition
STT: Spinothalamic tract
SRT: Spinoreticular tract
MR: Magnetic resonance
