# Supplementary material for: Cardiac cephalalgia: a narrative review and ICHD-3 criteria evaluation
Source: J Headache Pain. 2022 Oct 20;23(1):136. doi: 10.1186/s10194-022-01508-7 (PMC9583508; doi:10.1186/s10194-022-01508-7)
Supplement: Supplementary file 1 — Additional file1. Cardiac cephalalgia case reports. Summary of data from previous case reports of cardiac cephalalgia. [file 10194_2022_1508_MOESM1_ESM.pdf]

Author, year	Sex/age	Risk factors	Headache history	Location	Quality	Intensitiy	Nausea	Photophobia or phonophobia	Aggravated by exertion	Symptoms of cardiac ischemia	ECG	Cardiac enzyme	Stress test	Coronary angiogram	Treatment
Lefkowitz, 1982[7]	M/62	NS	NS	Bregmatic	Explosive	Severe	NS	NS	Yes	Retrosternal pain, numbness both arms	Chronic anterolateral ischemia	NS	Headache and ST depression	Triple-vessel disease	CAB
Fleetcroft, 1985[8]	F/78	NS	NS	Frontal to mouth	NS	NS	NS	NS	Yes	Chest pain radiating to both arms	ST elevation II, III, aVF	NS	NS	NS	Medical
Blacky, 1987[9]	M/40	No	NS	Bitemporal	NS	NS	No	NS	Yes	None	NS	NS	Headache and inferolateral ST-segment depression	100% CXA, severe stenosis RCA	Medical
Vernay, 1989[10]	M/71	Smoking	NS	Occipital, parietal and frontal	NS	NS	NS	NS	Yes	Shoulder numbness, subesteral tightness	Q wave in III and chronic anteroapical ischemia	NS	Headache and ST depression	NS	Medical
Takayanagi, 1990[11]	M/67	NS	NS	Occipital	NS	Severe	NS	NS	NS	Chest pressure	ST elevation/vent	NS	NS	NS	Death

											ricular fibrilation				
	F/64	CAD	NS	NS	NS	NS	NS	NS	NS	Chest pressure	ST elevation and Q waves in II, III	NS	NS	Triple-vessel disease (95% LAD, 99% RCA)	Death
Bowen, 1993[12]	M/59	HT, obesity, HL, smoking	Yes	Bitemporal	NS	Severe	No	No	NS	Left arm and chest pressure	ST Depression in anterior leads	NS	NS	100% RCA, 95% CXA	PTCA
Ishida, 1996[13]	M/64	Smoking	No	Occipital	Throbbing	Severe	Yes	NS	No	Bilateral shoulder pain	Q and inverted T waves in inferior leads	↑	Headache and ST-segment depression	Triple-vessel disease (99% RCA, 75% CXA)	PTCA
Grace, 1997[14]	M/59	NS	No	Vertex and occipital	Bursting	Severe	NS	NS	Yes	No	T wave inversion in aterolateral leads	NS	Headache with ST depression	Subocclusion RCA, 70% LDA	CAB
Lipton, 1997[3]	M/57	Past smoking	NS	Vertex	sharp	Severe	Yes	No	Yes	Chest disconfornt	Flattened T waves in II, and aVL, an inverted T wave in V_5, and biphasic T waves in III, V_4 and V_6	NS	Headache and ST depression in leads II, III, aVF, and V_{4-6}	Triple-vessel disease	CAB

	M/67	HT, past smoking	NS	Bifrontal	Pressing	Severe	No	No	Yes	No	Possible old inferior wall myocardial infarct	NS	Headache and inferolateral ST depression	Triple-vessel disease	PTCA
Lance, 1998[15]	M/62	Smoking, HL	No	Right frontal	NS	NS	NS	NS	Yes	Chest tightness	NS	NS	Headache and ST depression	100% LAD, 100% RCA	CAB
Lanza, 2000[16]	M/68	Smoking, HL	NS	Occipital	NS	NS	NS	NS	NS	Bilateral shoulder pain	Normal	↑	Headache and T wave peaking in V_{2-4}	Triple-vessel disease (90% LAD, 90% CXA, 100% RCA)	CAB
Rambihar, 2001[17]	F/56	NS	NS	Occipital	NS	NS	NS	NS	Yes	Shoulders and left arm	ST depression I, II, aVF, and V_4-V_6	NS	Headache and ST depression in I, II, sVF, and $V_{4–6}$	40% LM, 90% CXA, 99% RCA	CAB
	F/NS	NS	NS	Occipital	NS	NS	NS	NS	NS	None	NS	NS	Positive	NS	Death
Amendo, 2001[18]	F/78	HT, HL	NS	Bitemporal	NS	Severe	Yes	NS	NS	NS	ST elevation in I and aVL	↑	NS	Triple-vessel disease	CAB
	F/77	No	No	Right frontal and maxillary	NS	Severe	NS	NS	NS	None	T inversion	↑	NS	Normal coronary arteries	NS
Auer, 2001[19]	M/47	Smoking, HL	NA	Occipital	NS	NS	NS	NS	NS	NS	ST elevation	NS	NS	Autopsy: 100%	Death

											in I and aVL and ST depression in II, III, and aVF / Ventricular fibrilation			LAD, 80% RCA	
Famularo, 2002[20]	M/70	HT, smoking, stroke	No	Bilateral frontoparietal	Sharp	Severe	No	No	NS	Sweating	ST elevation and T-wave inversion V_2-V_3 and aVF	↑	Normal stress	NS	Medical
Guitiérrez-Morlote, 2002[21]	M/59	HT, HL, CAD	No	Bilateral, occipital and vertex	Dull, throbbing	Moderate - Severe	Yes	Yes	NS	Chest disconfort and sweating	ST-segment depression	NS	NS	NS	Medical
Martínez, 2003[22]	F/68	HT, DM, smoking	No	Hemicranial and facial	Shooting	Severe	No	No	Yes	None	Inverted T waves in V_2-V_4	NS	Headache and anterior, lateral, and septal ST depression	Triple-vessel disease	PTCA
Sathirapanya, 2004[23]	M/58	TIA, smoking	No	Left occipital	Sharp, shooting	Severe	No	No	Yes	Chest tightness	ST-T elevation V_1–V_3	NS	Positive	Triple-vessel disease	CAB
Chen, 2004[6]	M/76	HT	NS	Bitemporal	NS	Severe	No	No	Yes	Chest tightness	inverted T waves in leads I and AVL	NS	Headache and ST depression in II, III, and aVF	90% LAD, 50% RCA	PTCA and Medical

Gutiérrez-Morlote, 2005[24]	F/74	Stroke, DM, obesity	No	Bitemporal	Tthrobbing	Severe	Yes	NS	NS	Chest tightness, sweating	ST depression in V_2-V_4	↑	NS	NS	Medical
	F/64	HT, HL, obesity, DM	No	Unilateral, radiation to jaw	Pressing	Severe	NS	NS	Yes	No	NS	NS	NS	NS	Death
Korantzopoulos, 2005[25]	F/73	HT, HL, obesity	No	Occipital	Sharp	Severe	Yes	NS	NS	No	ST depression V_2-V_5	↑	Normal	70% LAD	Medical
Cutrer, 2006[26]	M/55	HT, HL, obesity, past smoking	No	Bilateral parietal, radiation to the jaws	NS	NS	No	No	Yes	No	NS	NS	Inferior ischemia	100% RCA, 60% LAD	PCTA
Seow, 2007[27]	M/35	Smoking	Yes	NS	Explosive	Severe	Yes	No	NS	Sweating	ST elevation V_{1-4}	↑	NS	100% LAD	PTCA
Broner, 2007[28]	F/72	No	No	Bi-occipital, radiating to bi-frontal region	Sharp, throbbing	Severe	Yes	No	No	No	ST elevatio II, III and avF and depression in I, avL, V_1 and V_2	↑	NS	100% RCA	Medical
Wei, 2007[29]	M/36	Stroke	NS	Vertex, occipital bilateral	Dull	Severe	NS	NS	NS	Cardiac arrest	ST segment elevation V_2-V_6	NS	NS	90% LAD	PTCA
	F/85	None	NS	Right eyeball pain	NS	NS	NS	NS	NS	Chest pain	NS	NS	NS	Normal coronary arteries	Medical

														(Apical hypertrophy)	
Wang, 2008[30]	F/81	HT	NS	NS	NS	Severe	Yes	NS	NS	Diaphoresis, cardiac arrest	Ventricular fibrilation	NS	NS	99% CXA	PTCA
Dalzell, 2009[31]	F/44	Smoking	NS	Occipital	NS	Severe	Yes	NS	NS	No	Inferior ST elevation and ST-depression in leads V_2-V_3	↑	NS	100% RCA	PTCA
Sendovski, 2009[32]	F/61	HT, HL	NS	Frontal, holocranial, neck, upper back, jaw and teeth	NS	Severe	No	No	Yes	No	ST depression at the lateral wall and a mild ST elevation at the precordial leads	↑	NS	70% LAD, 80% RCA, 95% CXA, 90% PDA	PTCA
Chatzizisis, 2010[33]	M/42	None	NS	Frontal, bitemporal	NS	Severe	NS	NS	NS	No	Q Wave, mild ST elevation and inverted T wave in V_2-V_5	↑	NS	100% LAD	PTCA
Cheng, 2010[34]	F/52	NS	NS	Bilateral	Throbbing	Severe	Yes	NS	Yes	Chest pain	Normal	↑	NS	Normal coronaries (suspected vasospasm)	Medical
	F/67	HT, DM, HL	No	Bilateral, temporoparietal and jaws	Throbbing	Severe	No	NS	Yes	Dyspnea	Normal	NS	NS	Two-vessel disease	PTCA

Yang, 2010[35]	F/44	No	NS	Bilateral, frontal, parietal and occipital	NS	Severe	No	No	Yes	Chest tightness	Normal	NS	Headache and ST depression	RCA vasospasm	Medical
Costopoulos, 2011[36]	M/55	HT, HL, smoking, DM	NS	Occipital to vertex	NS	NS	NS	NS	Yes	Breathlessness	Widespread ST depression	↑	NS	Triple-vessel disease	CAB
Elgharably, 2013[37]	M/55	Past smoking	No	Frontal	NS	Severe	No	No	NS	Chest pain	Q wave in III and aVF	↑	NS	LAD	PTCA
Asvestas, 2014[38]	M/86	HT, smoking	NS	Occipital	NS	Severe	NS	NS	NS	No	ST depression in V_1-V_5 and ST elevation in V_7-V_9	↑	NS	Triple-vessel disease (100% CXA, LAD, RCA)	PTCA
Wassef, 2014[39]	M/44	HL	NS	NS	Pressing	Severe	NS	NS	Yes	Chest disconfort	Normal	NS	Headache and lateral ST segment depression	100% LAD	PTCA
Mathew, 2015[40]	M/42	HT	Yes	Throbbing, bioccipital to vertex	NS	Variable	No	No	Yes	Palpitations and shortness of breath	NS	NS	Positive	99% LAD	PTCA

Chowdhury, 2015[41]	M/51	HT	NS	Temporal, frontal, vertex and occipital	NS	NS	No	No	Yes	Chest tightness, sweating	NS	NS	Headache and ST-T changes	70% LAD, 95% CXA	PTCA
Prakash, 2016[42]	M/67	Smoking	NS	Holocranial	NS	Severe	Yes	No	Yes	No	Q in II, III, aVF	NS	Headache and ST depression in inferior leads	Triple-vessel disease (90% RCA, 75% LAD, 70% CXA)	CAB
Huang, 2016[43]	F/70	HT, DM, HL	Yes	Bilateral neck pain and temporal	Dull, squeezing	NA	No	No	NS	Dizziness	ST-elevation V_2-V_6	↑	NS	LAD	PTCA
Shankar, 2016[44]	M/73	DM, stroke	No	Holocranial	Dull	severe	NS	NS	Yes	Chest pain	inferior-lateral ST depression	NS	Headache and ST depression in the inferior-lateral leads	Triple-vessel disease	CAB
Wang, 2017[45]	M/40	Past smoking	NS	Bitemporal	Pulsatile	Moderate	No	No	Yes	Chest disconfort, palpitations, sweating	Inverted T wave	Normal	NS	Triple-vessel disease (100% LAD, 100% RCA, 80% CXA)	PTCA
Majumder, 2017[46]	F/48	DM	NS	NS	NS	Severe	NS	NS	Yes	NS	ST depression with T-wave inversion in inferior leads	↑	NS	LAD, RCA	PCTA

MacIsaac, 2019[47]	M/86	No	NS	Bilateral holocrainal	Dull	Severe	No	No	No	Chest heaviness and dyspnea	Normal	Normal	Inferolateral ST depression	Triple-vessel disease (90% RCA, 50% LAD, 60% CXA)	Medical
Lazari, 2019[48]	M/64	Smoking	Yes	Holocranial	Compressing	Severe	NS	NS	NS	No	ST elevation II, III and aVF	NS	NS	RCA	PTCA
Santos, 2019[49]	M/62	HT, DM	No	Holocranial	NS	Severe	Yes	No	No	Chest pain	no ST elevation	↑	NS	Triple-vessel disease	CAB
Ruiz Ortiz, 2020[50]	F/78	HT, DM, HL	NS	Vertex, bifrontal, laterocervical	Compressing	Moderate	Yes	No	Yes	No	ST lateral and inferior	↑	NS	Triple-vessel disease	PTCA
Sun, 2021[51]	F/83	No	NS	NS	NS	NS	NS	NS	NS	No	ST elevation II, III, aVF, V_7-V_9	↑	NS	RCA	PTCA
Kobata, 2022[52]	M/69	Smoking, CAD	NS	Occipital	Pulsatile	Severe	Yes	NS	NS	Chest disconfort, sweating, loss of consciousness	ST elevation in II, III, aVF	↑	NS	RCA	PTCA
Kobata, 2022[52]	F/66	No	NS	Occipital	NS	Severe	NS	NS	NS	Chest disconfornt, sweating	ST elevation in II, III, aVF, V2-4	↑	NS	LAD	PTCA
Kobata, 2022[52]	M/54	Smoking, HT,	NS	Occipital	Strangulation	Moderate	Yes	NS	NS	Chest disconfornt, sweating	ST elevation in II, IIi, aVF	↑	NS	Triple-vessel disease	PTCA

		DM, HL													
Kobata, 2022[52]	M/72	Smoking, HT	NS	NS	NS	Severe	Yes	NS	NS	No	ST elevation in aVR, ST depression in II, III, aVF and V2-5	↑	NS	Triple-vessel disease	PTCA

M: Male; F: Female; NS: Not specified; CAB: Coronary artery by-pass; CXA: Circumflex artery; RCA: Right coronary artery; CAD: Coronary artery disease; HT: Hypertension; HL: Hyperlipidemia; PTCA: Percutaneous transluminal coronary angioplasty; LAD: Left anterior descending artery; PDA: posterior descending artery; DM: Diabetes mellitus; LM: Left main; TIA: transient cerebral ischemic attack.
